# Effect of RF Power on the Physical Properties of Sputtered ZnSe Nanostructured Thin Films for Photovoltaic Applications

**DOI:** 10.3390/nano11112841

**Published:** 2021-10-25

**Authors:** Ovidiu Toma, Vlad-Andrei Antohe, Ana-Maria Panaitescu, Sorina Iftimie, Ana-Maria Răduţă, Adrian Radu, Lucian Ion, Ştefan Antohe

**Affiliations:** 1 Faculty of Physics, R&D Center for Materials and Electronic & Optoelectronic Devices (MDEO), University of Bucharest, Atomiştilor Street 405, 077125 Măgurele, Romania; thtoma72@yahoo.com (O.T.); vlad.antohe@fizica.unibuc.ro (V.-A.A.); anamaria.pam11@yahoo.ro (A.-M.P.); sorina.iftimie@fizica.unibuc.ro (S.I.); ana.raduta@fizica.unibuc.ro (A.-M.R.); adrian.radu@fizica.unibuc.ro (A.R.); lucian@solid.fizica.unibuc.ro (L.I.); 2 Institute of Condensed Matter and Nanosciences (IMCN), Université catholique de Louvain (UCLouvain), Place Croix du Sud 1, B-1348 Louvain-la-Neuve, Belgium; 3 Academy of Romanian Scientists, Splaiul Independenţei 54, 050094 Bucharest, Romania

**Keywords:** zinc selenide (ZnSe), thin films, radio frequency (RF) magnetron sputtering, physical properties, spectroscopic ellipsometry, electrical measurements

## Abstract

Zinc selenide (ZnSe) thin films were deposited by RF magnetron sputtering in specific conditions, onto optical glass substrates, at different RF plasma power. The prepared ZnSe layers were afterwards subjected to a series of structural, morphological, optical and electrical characterizations. The obtained results pointed out the optimal sputtering conditions to obtain ZnSe films of excellent quality, especially in terms of better optical properties, lower superficial roughness, reduced micro-strain and a band gap value closer to the one reported for the ZnSe bulk semiconducting material. Electrical characterization were afterwards carried out by measuring the current–voltage (I-V) characteristics at room temperature, of prepared “sandwich”-like Au/ZnSe/Au structures. The analysis of I-V characteristics have shown that at low injection levels there is an Ohmic conduction, followed at high injection levels, after a well-defined transition voltage, by a Space Charge Limited Current (SCLC) in the presence of an exponential trap distribution in the band gap of the ZnSe thin films. The results obtained from all the characterization techniques presented, demonstrated thus the potential of ZnSe thin films sputtered under optimized RF plasma conditions, to be used as alternative environmentally-friendly Cd-free window layers within photovoltaic cells manufacturing.

## 1. Introduction

There is currently a high drive to develop semiconducting materials with easily-tunable properties that allow improved light-matter interactions in order to expand the performance and functionality of various optoelectronic devices, such as: infrared-sensitive elements [1,2], light emitting diodes (LEDs) [3,4], or photovoltaic (PV) cells [5]. In this context, zinc selenide (ZnSe) is a very attractive material from A${}^{\mathrm{II}}$-B${}^{\mathrm{VI}}$ binary semiconducting compounds with unique physical properties, such as, large direct band gap of 2.67 eV (at room temperature), low optical absorption in visible and infrared regions, high refractive index, high electrical conductivity, very good photosensitivity, and it is environmentally friendly [6]. Consequently, there are already numerous reported applications based on ZnSe thin films, such as LEDs [7,8], infrared devices [9,10], diodes [11], photodetectors [12], lasers [13], sensors [14], and solar cells [15], to mention only a few. Various techniques have been used to prepare ZnSe thin films including vacuum thermal evaporation [16,17,18], chemical bath deposition [19,20,21], chemical vapor deposition [22], sintering [23], close-spaced sublimation [24], electrodeposition [25], molecular beam epitaxy [26], laser deposition [27], or RF magnetron sputtering [9,28,29]. Regarding the use of ZnSe thin films in solar cells technology, among the Cd-free buffer layers, ZnSe is one of the most important candidates to replace cadmium sulfide (CdS) as a window material for solar cells. Besides environment considerations, ZnSe wider band gap energy (around 2.7 eV) as compared to the commonly-employed CdS (around 2.4 eV) is leading to the possibility of improving the transmission of blue light radiation, and enhancing the photocurrent. Notably, ZnSe material also has a good lattice match with Cu(In,Ga)(S,Se)${}_{2}$, therefore there are numerous reports showing the use of ZnSe thin films as buffer material in different solar cell configurations, such as CIGS-based architectures (copper indium gallium selenide), DSSCs (dye-sensitized solar cells), or CdTe-based photovoltaic devices [29,30,31,32,33,34]. This paper presents the preparation of ZnSe thin films by radio frequency (RF) magnetron sputtering, since this deposition technique proved to ensure high quality chalcogen compounds-based films with excellent thickness uniformity, as demonstrated in our previous studies focused on the synthesis of CdS [35] and ZnSe [29] thin films, respectively. In this paper, we particularly investigated the influence of the RF sputtering power on the structural, morphological, optical and electrical properties of the prepared ZnSe thin films. To the best of our knowledge, there are yet limited reports focusing on the correlation between the RF plasma power and the physical properties of the obtained ZnSe thin films, which are all important to get ZnSe-based solar cells with an expectedly better performance. Moreover, our results are promising, as they pointed out the optimal preparation conditions for ZnSe thin films, to be further used as a more environmentally-friendly, Cd-free buffer or window layer within various optoelectronic devices.

## 2. Materials and Methods

The ZnSe thin films were deposited by RF magnetron sputtering onto optical glass substrates, using a commercial ZnSe target (PI-KEM). The frequency of RF generator was fixed at 13.56 MHz. The deposition chamber was first evacuated at $10^{-3}$ Pa, before admission of argon (Ar) gas up to the working pressure of 0.86 Pa. Prior to the deposition, the substrates were ultrasonically cleaned in acetone, isopropyl alcohol and deionized water for 15 minutes in each bath, and dried over gaseous nitrogen flow. Other deposition parameters used in the sputtering process of the ZnSe films were target-to-substrate distance of 8 cm, the substrate temperature of 220 °C and the sputtering time of 30 min, while the RF power was varied as 60 W, 80 W, 100 W and 120 W. Films with different thicknesses were thus obtained and they will be indicated as follows: ZnSe1 (60 W), ZnSe2 (80 W), ZnSe3 (100 W) and ZnSe4 (120 W). The structural features of the fabricated samples were determined by X-ray diffraction (XRD), with a Bruker D8 Discover equipment (using CuK${}_{\alpha 1}$ radiation at λ=1.5406 Å). The topography of the fabricated ZnSe films’ surface was analyzed by atomic force microscopy (AFM) in tapping mode, using an A.P.E. Research A100-SGS instrument. The acquired AFM micrographs have been afterwards post-processed in Gwyddion software package for extracting the roughness average ($R_{A}$), root mean square roughness (RMS), as well as Skewness (Ssk) and Excess Kurtosis (Sku) specific statistical parameters. Subsequently, cross-sectional morphological observations of the prepared ZnSe thin films was carried out by scanning electron microscopy (SEM) using a Tescan Vega XMU-II equipment, mainly to allow an overall direct evaluation of the films thickness. The optical properties of ZnSe thin films deposited on glass substrates were next investigated by optical spectroscopy (OS), i.e., transmission and absorption, using a spectrophotometer Perkin-Elmer Lambda 750, within the spectral range between 200 nm and 1200 nm, in air and at room temperature. Going further, optical constants (refractive indices and extinction coefficients) were also investigated using spectroscopic ellipsometry (SE) with a phase modulated spectro-ellipsometer (PME) from Horiba. The angle of incidence was 75° for all ZnSe samples while the modulation frequency of the photoelastic modulator was set at 50 kHz. Ultimately, the dark current–voltage (I-V) characteristics of the sandwich structures, i.e., Au/ZnSe/Au, were measured at room temperature using a computer-controlled experimental setup including a Keithley 6517a electrometer, a Keithley 2400 SourceMeter and a Lakeshore 332 temperature controller.

## 3. Results and Discussion

### 3.1. Structural Characterizations

**Figure 1:**
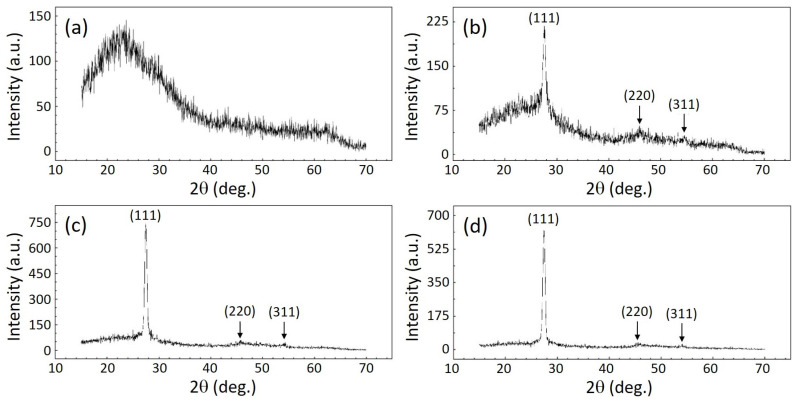
GIXRD patterns of the ZnSe thin films deposited by RF magnetron sputtering onto optical glass substrates at an RF power of: (**a**) 60 W, (**b**) 80 W, (**c**) 100 W, and (**d**) 120 W.

**Figure 2:**
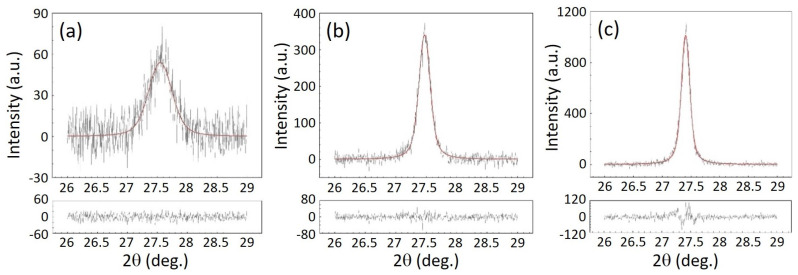
XRD profiles recorded for (111) peak in Bragg–Brentano theta–theta geometry for samples: (**a**) ZnSe2, (**b**) ZnSe3, and (**c**) ZnSe4.

The structural analysis was performed first in grazing incidence (GIXRD) geometry at an angle of 1°. This type of measurement provides a better signal from thin films and minimizes the signal from the substrate. In Figure 1, the results from GIXRD analysis are shown for ZnSe samples and one can observe that generally the crystalline structure is improved with increasing the RF power and thickness. In particular, when comparing the GIXRD patterns of ZnSe samples deposited at different RF powers (i.e., 60–120 W), it was found that the 60 W RF power was not sufficient to form a ZnSe crystalline layer (Figure 1a). This behavior can be understood in terms of the low energy atoms which are easily adsorbed on the glass substrate, moving over and interacting to form clusters, but without posing enough energy to overcome the nucleation barrier, hence to reach thermodynamic stability that favors the formation of ZnSe crystallites. In contrast, by increasing the RF power towards 120 W (Figure 1b–d), a reducing background noise and an increasing intensity peak reflected from the (111) planes of the ZnSe cubic structure could be observed, together with low intensity peaks emanating from the (220) and (311) plane orientations, respectively. Moreover, as described further, the analysis of the most intense peak (111)—Figure 2, also reveals improvements of the crystalline quality of the samples with increasing the RF power, as the narrowness of the reflected (111) peaks increases with RF power. This behavior could be explained by the fact that an increased RF power improves the electrons mobility, thus further the Ar gas atoms ionization efficiency. Consequently, the highly energized inert Ar ions provide translational kinetic energy to the adatoms sputtered on the growing surface, enhancing their surface diffusion that finally leads to obtaining films with high-quality crystalline structure. This approach was also used to explain a similar behavior in the case of other zinc blend-like crystalline structure materials [36,37].

Crystalline structural parameters of the samples were determined by analyzing in Bragg–Brentano theta–theta geometry the most intense reflected plane (111). This characterization is suitable for finding parameters, such as grain size ($D_{ef}$), micro-strain (${\langle \varepsilon^{2}\rangle }^{1/2}$) and lattice constant (*a*) that can be calculated using the information obtained after a proper evaluation of the data (see Table 1) [38]. Figure 2 presents the recorded profiles and the fitting curves with Voigt profiles, as well as the residuals of the experimental data after the processing using the theoretical model (the lower plots). It can be observed from Table 1 that when the RF power is increased, bigger crystallite size is obtained, with a small mean-square strain and a value for lattice constant which is closer to that found in bulk crystal [39], the ideal ZnSe lattice constant being $a_{0}=5.669$ Å (according to PDF2 37-1463 card). In particular, the crystallite size ($D_{ef}$) increased from 51.8 nm to 107.4 nm when raising the RF power, which is a predictable result taking into account the sharpening of the (111) peak as a result of a higher crystallinity, as can be observed within XRD patterns presented in Figure 2. It can be thus noticed from Table 1 that at higher thickness, with the relating sputtering parameters, the structural texture is improved. As explained above, when the nucleation barrier is overcame as a consequence of RF power increase, thermodynamically stabilized crystallites are formed, causing their size to become larger, until a saturation of the nucleation density occurs, causing the ZnSe crystalline layers formation, and determining further a natural increase of the films thickness and so of the crystallites size with RF power, as observed in Table 1.

### 3.2. Morphological Characterizations

**Figure 3:**
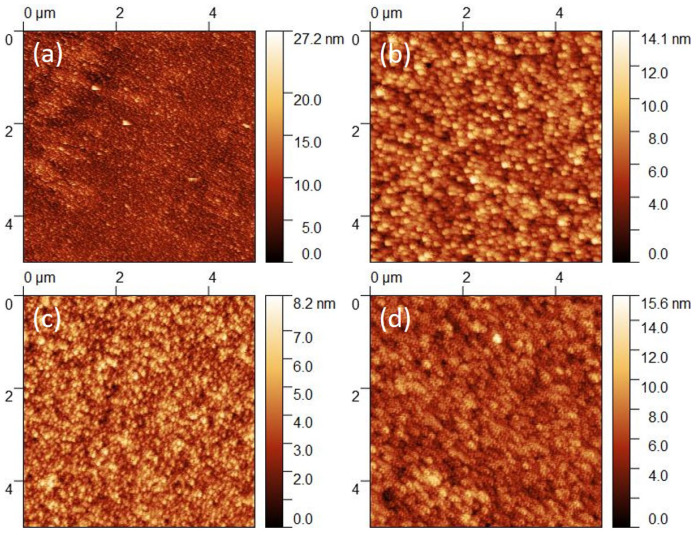
AFM 2D surface topography images of the RF-magnetron sputtered ZnSe thin films, prepared at an RF power of: (**a**) 60 W, (**b**) 80 W, (**c**) 100 W, and (**d**) 120 W.

The surface topography of the fabricated ZnSe samples was analyzed by AFM, in tapping mode. The 2D AFM images of the surface topography of the grown ZnSe thin films are shown in Figure 3, while the evaluated morphological parameters are summarized in Table 2. For all samples, the scanned area was 5×5 μm${}^{2}$. As can be noticed from Figure 3, all samples present good uniformity of the ZnSe thin films and a granular surface, with homogenously arranged grains, that slightly changes with RF power. Notably, the overall Z-range reduces with increasing RF power towards 100 W (see Figure 3a–c), then it raises a little at 120 W (see Figure 3d). The latter remark is confirmed by the calculated average roughness ($R_{A}$) and root mean square roughness (RMS) parameters (see Table 2), which are decreasing, i.e., from 1.9 nm and 2.4 nm, respectively, at 60 W, to 0.8 nm and 1.0 nm, respectively, at 100 W, proving that for this RF power domain the sticking coefficient is increased by the increase of the kinetic energy when the ionized atoms hit the substrate [37,40]. Notably, this observation is also strengthened by the increase of the crystallite size with increasing the RF power (see Table 1), confirming that in given conditions, for a certain range of RF power, the crystallite size raises, while the surface roughness decreases with the sputtering power. This trend is consistent with previous studies, mentioning that at an exceedingly high RF power, the surface roughness could increase due to excessive scattering effects close to the substrate’ surface that typically reduce the overall sputtering rate and favor the formation of defects and coarse grains at the surface of the sputtered films [41], effect observed in our studies, too, by an increase of $R_{A}$ and RMS values, i.e., to 1.3 nm and 1.6 nm, respectively, for the ZnSe film deposited at 120 W (see Table 2). Notably, the ZnSe film grown at 100 W (ZnSe3) features the smallest $R_{A}$ and RMS values, i.e., 0.8 nm and 1.0 nm, respectively, in respect to the rest of the fabricated samples, eventually demonstrating that the latter sputtering conditions could allow the fabrication of ZnSe thin films with lowest defects density and excellent flatness, suitable to be used as “window” or buffer layers within various solar cells architectures.

The smoothness of the prepared ZnSe films has also been interpreted in terms of the determined statistical parameters, i.e., Skewness (Ssk) and Excess Kurtosis (Sku) coefficients (see Table 2), which generally indicate clean and almost flat surfaces of all the sputtered ZnSe films. In particular, an overall positive Skewness with Ssk values close to zero (i.e., 0.3), clearly indicates the presence of a very limited number of height values above the average, although the film grown at 60 W could exhibit a flat surface with tiny protruding features as the slightly larger SSk value of 0.8 suggests. Complementarily, the calculated Sku coefficients statistically suggest a distribution of the heights with a Platykurtic profile of the examined surfaces (Ssk < 3), hence with a small variation of the heights in respect to the horizontal reference plane. As one can easily notice, the sample prepared at 60 W exhibits a higher Sku value (i.e., 1.4), confirming the hypothesis of presenting few extreme protruding heights on the surface. In contrast, the ZnSe film sputtered at 100 W features the lowest Sku value (i.e., 0.1), pointing out again that in this conditions the ZnSe film surface is homogenous and flat without any pit or hillock defects [42,43].

**Figure 4:**
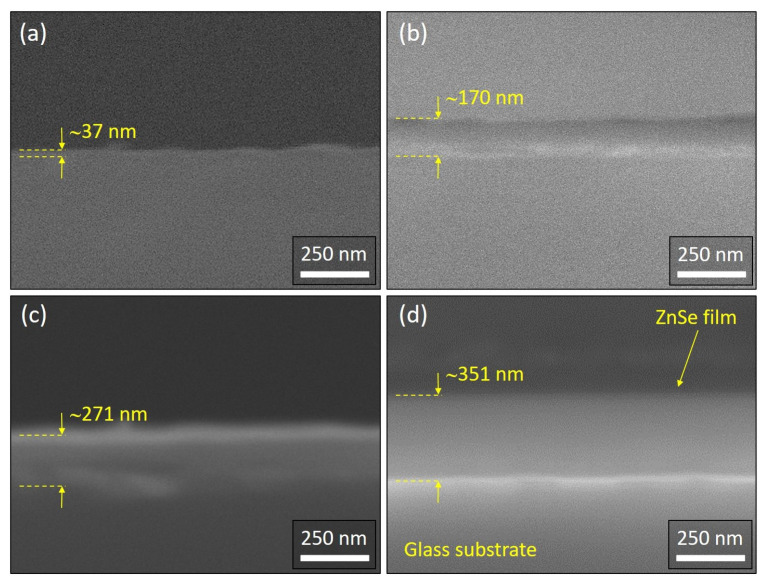
Cross-sectional SEM micrographs of the ZnSe thin films sputtered on glass substrates for 30 min, while keeping a constant gas pressure of 0.86 Pa and applying an RF power of: 60 W (**a**), 80 W (**b**), 100 W (**c**) and 120 W (**d**). Corresponding SEM measurements of the ZnSe films thickness are indicated.

**Figure 5:**
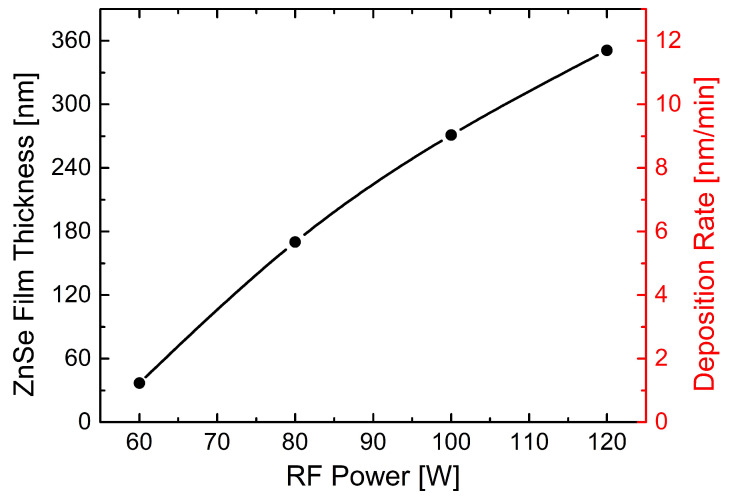
Variation of ZnSe films thickness with RF power, in given sputtering conditions as described in the text. The curve is based on the SEM analysis of the samples. Corresponding calculated sputtering rate is indicated in nm/min on the right red Y-axis.

Cross-sectional observations of the ZnSe-coated glass substrates were performed by SEM operating in secondary electron imaging mode. The SEM specimens were carefully prepared by dicing the ZnSe–sputtered glass substrates and partially covering their section with conducting carbon tape to minimize the charging effects, hence to facilitate the SEM analysis. Figure 4 shows cross-sectional SEM micrographs of the ZnSe thin films deposited on glass by magnetron sputtering in an RF plasma engaged in Ar atmosphere kept at 0.86 Pa for 30 min, at different RF powers of 60 W (a), 80 W (b), 100 W (c) and 120 W (d). Several measurements have been acquired for each sample to allow an estimation of the films thickness, as indicated in the images. Average thickness values, as estimated from the SEM analysis, are collected in Table 2. It can be observed that the obtained ZnSe films are compact and sputtered on the glass substrates with good conformality. Experimental data resulted from the SEM analysis are graphically represented in Figure 5, where the black circles represent the SEM-measured thickness values, while on the right red Y-axis, the corresponding calculated sputtering rate is shown. As can be noticed, the ZnSe thickness increases with RF power, while the deposition rate slows-down towards 120 W. The observed behavior in Figure 5 can be obviously attributed to an increase of the kinetic energy and velocity of the particles being sputtered away from the target which results in a higher deposition rate [44]. However, the slowing-down of the increase in the deposition rate observed towards 120 W can be associated with an excessively high energy of the ions injection into the target, leading to an energy and quantity loss of the Ar ions, thus causing further a reduction in the deposition rate, typical behavior also mentioned elsewhere [45].

### 3.3. Optical Characterizations

**Figure 6:**
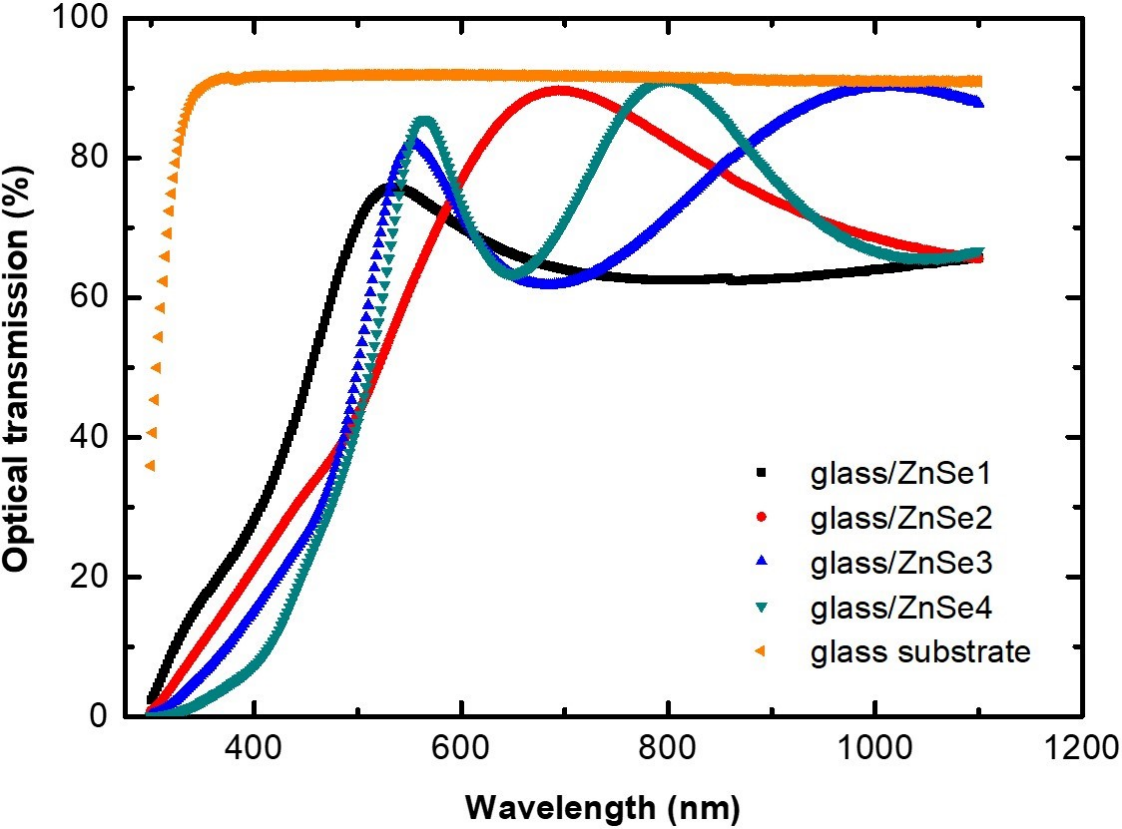
Optical transmission spectra of ZnSe thin films deposited onto optical glass substrates at different RF power. The reference spectrum of the optical glass substrate is depicted in orange.

Optical properties of ZnSe thin films deposited on glass substrates were investigated by optical transmission and absorption spectroscopy in the spectral range between 200 nm and 1200 nm at room temperature. The optical transmission results are shown in Figure 6. As a reference, the optical transmission of the glass substrate is also presented (the orange curve). Except for the sample ZnSe1, all investigated films show high transmittances in the visible range, with values larger than 80%. The reason behind a reduced optical transmission of the ZnSe film sputtered at 60 W, could be related to its inferior morphological and structural properties, as suggested by both the GIXRD pattern presented in Figure 1a and the AFM parameters collected in Table 2.

**Figure 7:**
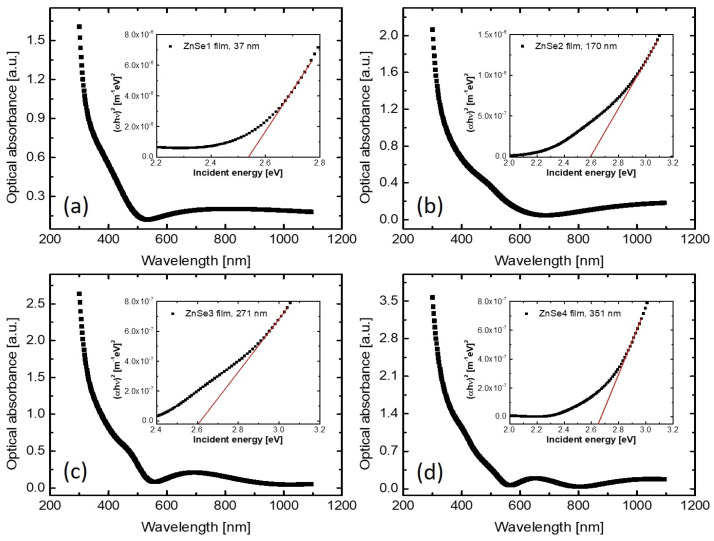
Spectral dependencies of optical absorbance for samples ZnSe1 (**a**), ZnSe2 (**b**), ZnSe3 (**c**), and ZnSe4 (**d**). The inset of graphs shows the ${\left(\alpha \hbar \omega \right)}^{2}$ *vs.* ℏω dependencies (Tauc’s plots), used to determine the optical band gap energies ($E_{g_{\left(OS\right)}}$) of the films.

Figure 7 shows the acquired optical absorbance data of the samples ZnSe1 to ZnSe4. The calculation of the absorption coefficient was performed using the following expression:

(1) $$\alpha =\frac{A}{d}$$

where *A* is the optical absorbance and *d* is the thickness of the films. The thickness values that were considered for the computation of the optical band gap of the prepared RF-sputtered ZnSe films were those determined by cross-sectional SEM analysis (see Table 2). Later on, α was used to estimate the optical band gap energy using the Tauc’s plot method following the well-known relationship:

(2) $$\alpha =A\frac{{\left(\hbar \omega -E_{g}\right)}^{1/2}}{\hbar \omega }$$

Equation (2) gives the dependence of the absorption coefficient on incident photons energies in the case of direct band gap semiconductors (like ZnSe) near the fundamental absorption edge, in which α is the absorption coefficient, *A* is a constant, ℏω is the energy of incident photons and $E_{g}$ is the optical bandgap corresponding to Γ point in the first Brillouin zone. The insets of Figure 7a–d present the corresponding Tauc’s plots, where the values for the optical band gap energies can be determined by the interceptions over the energy axis of the extrapolated linear portion of the plots. The values of the band gap energies of ZnSe films ($E_{g_{\left(OS\right)}}$) as obtained by fitting of the experimental data using Equation (2) are collected in Table 3. The obtained results are similar to those from literature for ZnSe thin films deposited by vacuum thermal evaporation [46].

**Figure 8:**
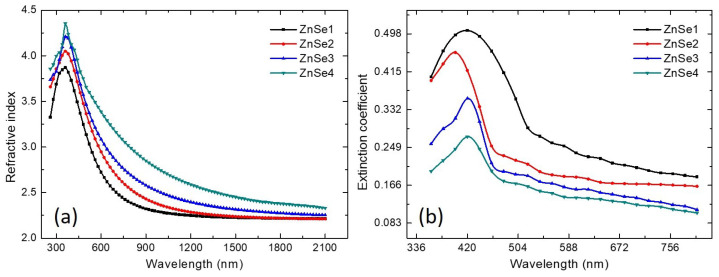
Spectral dependencies of refractive indices (**a**) and extinction coefficients (**b**) of the ZnSe thin films with different thicknesses, prepared by RF magnetron sputtering while varying the RF power.

Optical constants (refractive index and extinction coefficient) of ZnSe thin films deposited on optical glass substrates were determined using SE. The ellipsometric (Ψ, Δ) spectra were recorded in reflected light at an incident angle of 75° with respect to film surfaces. An optical three layers model (upper rough layer/ZnSe layer/glass substrate) was used while the dispersion model chosen for fitting the ellipsometric spectra was Adachi–New Forouhi (ANF) model [47]. After choosing the optical model and the dispersion function, the fitting parameters were varied by least square regression until a minimum difference between experimental and computed (Ψ, Δ) spectra was obtained. As a risk function, the mean-squared error (MSE) was used and the Levenberg–Marquardt regression algorithm was used in order to minimize MSE function [48]. Spectra of refractive indices (*n*) and extinction coefficients (*k*) of the films are plotted in Figure 8, whilst the results obtained by SE are displayed in Table 4. One can observe that the refractive index (Figure 8a) tends to increase with increasing the thickness of the films. The decrease of the refractive index with the increase of photon wavelength in the visible region (VIS) of the electromagnetic spectrum indicates the normal dispersion behavior of ZnSe thin films in this region. In near-infrared (NIR) region the refractive index tends to be relatively constant, the obtained values being in good agreement with previous works [49,50]. Due to the fact that ZnSe films are almost transparent in NIR region, extinction coefficients were displayed in Figure 8b only for VIS region, their values increasing with photon energy. Increasing the thickness of ZnSe films by increasing the RF sputtering power leads to a decrease in extinction coefficients. The values of the extinction coefficients obtained from the SE. data analysis, were then used to compute the absorption coefficients, using the commonly known equation:

(3) $$\alpha =\frac{4\pi k}{\lambda }$$

where α is the absorption coefficient, *k* is the extinction coefficient and λ represents the wavelength.

Further, knowing the absorption coefficients, Tauc’s method was similarly used to compute the energy band gap values ($E_{g_{\left(SE\right)}}$) using Equation (2). Notably, the values of band gap energies, showing a shift from the standard bulk band gap value of 2.7 eV (Table 4), match very well the values calculated from OS analysis of the ZnSe thin films (Table 3). The observed tendency of the band gap increase with the raise of the RF power can be explained by the domain of nanocrystallite sizes, ranging from 51.8 nm to 107.4 nm when the RF power is increased from 80 to 120 W, respectively (see Table 1), together with a reduction of the number of inter-grain boundaries and an increase of the thin film thickness. Taking into account these changes induced by increasing the RF power, the density of defects, leaving-out energy levels in the semiconductor’s band gap and thus being involved in the value of the optical band gap, reduces within whole grains and especially at their central parts. Hence, the optical band gap tends to increase towards the corresponding value of the bulk single crystal semiconductor, here 2.7 eV [51]. As can be seen in Table 4, relatively small values for MSE function were obtained for all investigated ZnSe samples indicating that the fitting procedure of ellipsometric spectra was performed accurately.

### 3.4. Electrical Characterizations

**Figure 9:**
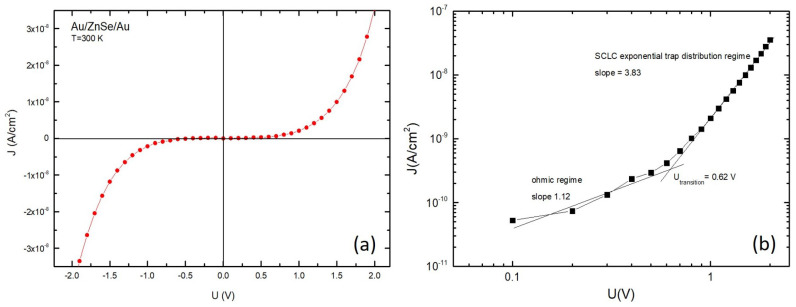
(**a**) Current–voltage (I-V) dark ambipolar characteristics recorded at room temperature for Au/ZnSe/Au sandwich structure, with ZnSe thin film sputtered at 100 W. (**b**) Corresponding I-V characteristics of Au/ZnSe/Au at room temperature, plotted in a double-logarithmic scale.

Ultimately, Au/ZnSe/Au sandwich structures were fabricated via RF magnetron sputtering of ZnSe at 100 W, by using Au electrodes in order to diminish the noise from contacts. The current–voltage (I-V) dark characteristic at room temperature (300 K) is presented in Figure 9a, showing a nonlinear but symmetrical behaviour, as expected when using the two electrodes made of the same metal. Figure 9b depicts the characteristics in logarithmic scale of two linear fits having different slopes, suggesting a transition from one conduction mechanism to another. At low injection levels (low applied voltage-LV) the slope of the first linear fit is around 1 (slope = 1.12) leading to the assumption that the device enters an ohmic conduction regime described by the following equation [52,53]:

(4) $$J_{ohmic}=qn_{0}\mu \frac{U}{d}$$

where *q* is the electronic charge, $n_{0}$ is the concentration of thermally generated free electrons in the conduction band at thermal equilibrium, μ is the electron mobility, *U* is the applied voltage and *d* is the thickness of ZnSe film (considered 271 nm for the sample sputtered at 100 W). In the LV regime, the transport through the structure is thus realized by the thermal equilibrium charge carriers of $n_{0}$ concentration.

With an evaluated value of 0.6 V for the transition voltage ($U_{\mathrm{transition}}$), the aforementioned structure has at high injection levels (HV) a slope of 3.83 (Figure 9b), suggesting, as expected for high resistivity materials [54,55], the existence of a conduction mechanism of space charge limited currents (SCLC). The analytical relationship describing the I-V characteristics in the HV regime over $U_{\mathrm{transition}}$, is frequently a power function but not always, because it generally depends on the presence of the defect levels in the band gap of the semiconductor, hence changing as a function of defects concentration and their energy distribution [56]. Taking into account the slope value (3.83), in the case of the prepared ZnSe thin films, there is a SCLC in the presence of an exponential trap distribution described by:

(5) $$\rho \left(E\right)=\frac{N_{t}}{k_{B}T_{C}}e^{\frac{-E}{k_{B}T_{C}}}$$

where ρ(E) is the trap density per unit energy range at an *E* level of energy below the conduction band, $N_{t}$ is the total density of trapping levels in the exponential distribution, $k_{B}$ is the Boltzmann constant and $T_{C}$ is the characteristic temperature which is a typical parameter for the exponential trap distribution showing how fast the variation of the density of traps is, with the changing of energy measured from the minimum of conduction band towards the middle of the band gap (in the case of traps for electrons). Based on the above-mentioned distribution, the I-V characteristics in the HV regime is described by a power function as it follows [53]:

(6) $$J_{SCLC_{exp}}=q\mu N_{C}{\left(\frac{\varepsilon }{qN_{t}}\right)}^{\gamma }\frac{U^{\gamma +1}}{d^{2\gamma +1}}\;\;,\;\;\gamma =\frac{T_{C}}{T}$$

where $N_{C}$ is the effective density of states in the conduction band, ε is the dielectric constant of ZnSe, γ is the ratio between $T_{C}$ (characteristic temperature) and *T* (absolute temperature, i.e., 300 K in this case). As one may see in Figure 9b, the linear fit, i.e., $\log_{10}J_{SCLC_{exp}}=f\left(\log_{10}U\right)$, in the HV region has a slope of 3.83 (γ+1), therefore the γ coefficient is 2.83 and the characteristic temperature $T_{C}$ is 849 K. Coupling thus the analytical relationship of the SCLC for an exponential trap distribution in the band gap of ZnSe, i.e., Equation (6), with the obtained experimental results, the above-mentioned parameters have been obtained through fitting the experimental data with the typical SCLC I-V characteristic of an exponential trap distribution. Such a methodological approach was already engaged in many other reports on similar materials [57,58], including our previous studies [16,32,35,42,52].

## 4. Conclusions

ZnSe thin films with different thicknesses were deposited onto optical glass substrates through RF magnetron sputtering, by varying the deposition power from 60 W to 120 W. The XRD structural characterization emphasizes the polycrystallinity of these thin films with a pronounced (111) texture, showing as well, an increase of the crystallite size with increasing the RF plasma power. Furthermore, the AFM superficial morphology analysis of the films indicates that while raising the RF power, $R_{A}$ and RMS parameters decrease, the ZnSe layer prepared at 100 W exhibiting an almost flat surface with the smallest values of roughness parameters, i.e., 0.8 nm and 1 nm, respectively. Additional cross-sectional SEM analysis allowed then a direct estimation of the ZnSe films thickness, showing as expected, an increase of the thickness by increasing the incident power, from 37 nm to 351 nm. In a second stage, OS (absorption and transmission) and SE techniques were employed to evaluate the band gap energies and to extract the optical constants of the ZnSe thin films (refractive indices and extinction coefficients). Notably, the band gaps estimated by both optical methods were similar, the values spanning between 2.54 eV and 2.65 eV, and showing a visible tendency of band gap increase with raising the RF power. Finally, the electrical measurements highlighted that the ZnSe thin films have high resistivity and at high-injection levels the conduction mechanism relies on a SCLC with an exponential trap distribution. The results obtained from all our investigations pointed out that in given sputtering conditions, at an RF plasma power of 100 W, the obtained ZnSe thin films exhibit superior properties, in terms of: better crystallinity, appropriate thickness maintaining a high transparency, low $R_{A}$ and RMS values, an adequate optical band gap of 2.61 eV (slightly larger value compared to the one of the conventional CdS layer), and ultimately good electrical properties. Consequently, these parameters are optimal to prepare excellent ZnSe thin films, perfectly suitable to be used (i) as environmentally-friendly “window” materials for solar cells to reduce the amount of Cd, commonly used for the second generation of solar cells relying on CdTe as main absorber, or alternatively, (ii) as buffer layers in solar cell architectures completely free of Cd.

## Figures and Tables

**Table 1:** Structural parameters of the ZnSe thin films deposited by RF magnetron sputtering at an RF power of: 80 W (ZnSe2), 100 W (ZnSe3), and 120 W (ZnSe4).

Sample	RF Power (W)	$D_{\mathrm{ef}}$ (nm)	${\langle \varepsilon^{2}\rangle }^{1/2}$	*a* (Å)
ZnSe2	80	51.8	$5.39\;\cdot \;10^{-3}$	5.603
ZnSe3	100	79.7	$2.31\;\cdot \;10^{-3}$	5.619
ZnSe4	120	107.4	$2.19\;\cdot \;10^{-3}$	5.628

**Table 2:** Morphological parameters evaluated by AFM in tapping mode for the ZnSe thin films RF-sputtered at: 60 W, 80 W, 100 W, and 120 W. Corresponding thickness values of the films, as measured by SEM, are indicated as well.

Sample	RF Power (W)	Thickness (nm)	$R_{A}$ (nm)	RMS (nm)	Ssk	Sku
ZnSe1	60	37	1.9	2.4	0.8	1.4
ZnSe2	80	170	1.5	1.8	0.3	0.2
ZnSe3	100	271	0.8	1.0	0.3	0.1
ZnSe4	120	351	1.3	1.6	0.3	0.5

**Table 3:** Calculated band gap ($E_{g_{\left(OS\right)}}$) values (from OS analysis) of the ZnSe thin films sputtered at an RF power of: 60 W, 80 W, 100 W and 120 W. Corresponding thickness values of the films, as measured by SEM, are indicated as well.

Sample	RF Power (W)	Thickness (nm)	$E_{g_{\left(\mathrm{OS}\right)}}$ (eV)
ZnSe1	60	37	2.54
ZnSe2	80	170	2.59
ZnSe3	100	271	2.60
ZnSe4	120	351	2.65

**Table 4:** Refractive indices (*n*) and extinction coefficients (*k*), as obtained from the SE analysis. Corresponding band gap ($E_{g_{\left(SE\right)}}$) values as calculated form the SE data and the SEM-measured thicknesses are indicated as well.

Sample	RF Power (W)	Thickness (nm)	*n* (at λ=600 nm)	*k* (at λ=600 nm)	$E_{g_{\left(\mathrm{SE}\right)}}$ (eV)	MSE
ZnSe1	60	37	2.723	0.237	2.56	6.02
ZnSe2	80	170	2.946	0.184	2.59	5.85
ZnSe3	100	271	3.083	0.157	2.61	6.81
ZnSe4	120	351	3.386	0.139	2.64	4.66

## Data Availability

Not applicable.

## Abbreviations

The following abbreviations are used in this manuscript:

R&D	Research & Development
MDEO	R&D Center for Materials and Electronic & Optoelectronic Devices
IMCN	Institute of Condensed Matter and Nanosciences
UCLouvain	Université catholique de Louvain
RF	Radio frequency
(GI)XRD	(Grazing incidence) X-ray diffraction
AFM	Atomic force microscope
SEM	Scanning Electron Microscope
OS/SE	Optical spectroscopy/Spectroscopic ellipsometry
PME	Phase modulated spectro-ellipsometer
2D	Two-dimensional
RMS	Root mean square roughness
Ssk/Sku	Skewness/Kurtosis
MSE	Mean-squared error
VIS	Visible spectral region
NIR	Near-infrared spectral region
SCLC	Space charge limited currents
UEFISCDI	Executive Unit for Financing Higher Education, Research, Development and Innovation
